# Long‐term experience of MPC across multiple TrueBeam linacs: MPC concordance with conventional QC and sensitivity to real‐world faults

**DOI:** 10.1002/acm2.12950

**Published:** 2020-08-13

**Authors:** Michael Pearson, David Eaton, Tony Greener

**Affiliations:** ^1^ Medical Physics Department Guys and St Thomas' Hospital London SE1 9RT United Kingdom

**Keywords:** automated QC, linac quality control (QC), linear accelerator, machine performance check (MPC), quality assurance

## Abstract

Machine Performance Check (MPC) is an automated Quality Control (QC) tool that is integrated into the TrueBeam and Halcyon linear accelerators (Linacs), utilizing the imaging systems to verify the Linac beam and geometry. This work compares the concordance of daily MPC results with conventional QC tests over a 3‐year period for eight Linacs in order to assess the sensitivity of MPC in detecting faults.

The MPC output measurements were compared with the monthly ionization chamber measurements for 6 and 10 MV photon beams and 6, 9, 12, 16, and 18 MeV electron beams. All 6 MV Beam and Geometry (6MVBG) MPC test failures were analyzed to determine the failure rate and the number of true and false negative results, using the conventional QC record as the reference. The concordance between conventional QC test failures and MPC test failures was investigated.

The mean agreement across 1933 MPC output and monthly comparison chamber measurements for all beam energies was 0.2%, with 97.8% within 1.5%, and a maximum difference of 2.9%. Of the 5000–6000 MPC individual test parameter results for the 6MVBG test, the highest failure rate was BeamOutputChange (0.5%), then BeamCenterShift (0.3%), and was ≤ 0.1% for the remaining parameters. There were 50 true negative and 27 false negative out of tolerance MPC results, with false negatives resolved by repeating MPC or by independent measurement. The analysis of conventional QC failures demonstrated that MPC detected all failures, except occasions when MPC reported output within tolerance, a result of the MPC–chamber response variation.

The variation in MPC output versus chamber measurement indicates MPC is appropriate for daily output constancy but not for the measurement of absolute output. The comparison of the 6MVBG results and conventional records provides evidence that MPC is a sensitive method of performing beam and mechanical checks in a clinical setting.

## 1 Introduction

The Quality Control (QC) programs of linear accelerators (Linacs) in the clinic generally follow the frequency, testing methods, and tolerances in published national guidance.^1^, ^2^ In recent years, automated QC programs utilizing the electronic portal imaging device have been proposed,^3^, ^4^ and Linac vendors have begun to incorporate QC applications into the Linac platforms. One such application is Machine Performance Check (MPC), an automated QC tool that is integrated into the Varian TrueBeam and Halcyon (Varian Medical Systems, Inc., Palo Alto, CA, USA). MPC utilizes a dedicated phantom with embedded ball bearings, and the kV and MV imaging systems to perform an independent check of beam output, uniformity, and various geometric parameters. The application automatically analyzes the kV and MV images that are acquired at a range of Gantry, Collimator, and Couch positions. MPC can be run daily during the Linac run‐up, and takes around 10 min to complete, dependent on the number of beam energies measured.

There have been a number of publications evaluating and validating the accuracy of MPC^5^, ^6^, ^7^, ^8^, ^9^, ^10^ since it was first introduced by Varian in 2015. The testing of MPC to date has been via two methods: either comparing constancy over time against another established measurement method^5^, ^6^, ^7^, ^8^, ^9^ or via the introduction of deliberate errors.^6^, ^7^, ^8^, ^10^ Methods for introducing deliberate errors included intentionally incorrectly calibrating a Linac parameter, for example, the Linac beam symmetry or MLC position calibration,^6^, ^7^, ^8^ or by introducing solid water into the beam path,^10^ or by applying known motions to the MPC phantom using a rotating/linear motion stage.^10^ The results of this testing of deliberate errors in mechanical and imaging parameters demonstrated sub‐mm and sub‐degree accuracy.^6^, ^7^, ^8^, ^10^ With regard to beam characteristics, MPC was able to detect a deliberate steering error in the 6 MV beam center to within 0.2 mm of the IC PROFILER (Sun Nuclear Corporation, Melbourne, FL, USA) ionization chamber array measurement, and the uniformity agreement with symmetry from the IC PROFILER was within 0.9% for 6 and 10 MV flattened and flattening filter free (FFF) beams.^6^ This testing demonstrates that MPC is capable of detecting beam and mechanical faults to a level that is appropriate for QC, comfortably less than the 1–2 mm and 2–3% tolerances in TG142^1^ and IPEM report 81v2.^2^

MPC results have been compared over time against the results from other established measurement methods, over periods of 3 weeks^5^ up to 1 year.^9^ Of these, the longest datasets are Barnes and Greer,^6^ which compared MPC on a single Linac over a 5‐month period for both flattened and FFF 6 and 10 MV beams, and Binny et al^9^ who report results for six TrueBeams over periods ranging 4.5–12 months (average 7.5 months) for 6 and 10 MV photon beams and 6, 9, 12, and 16 MeV electron beams. Barnes and Greer^6^ showed that the agreement between Farmer ionization chamber and MPC output measurements was 0.6% over the 5 months. Binny et al^9^ demonstrated that mean output variations were within ± 0.5% compared with Farmer ionization chamber and ± 1.5% compared with Daily QA3 (Sun Nuclear Corporation, Melbourne, FL, USA) dose constancy results, respectively.

This work aimed to evaluate MPC over a much longer period of time than has been reported previously, reporting on 3 yr of using MPC daily across eight Linacs, which equates to over 20 Linac years. We report the output stability for 6 and 10 MV photon beams, and 6, 9, 12, 16, and 18 MeV electron beams. In addition, rather than testing the sensitivity of MPC to deliberate errors, that has been reported previously, we instead evaluate the ability of MPC to detect real‐world QC faults over the 3‐year period by comparison with conventional QC testing records. We assess the sensitivity of MPC to detect faults in the clinical setting and therefore the suitability of MPC as a routine QC tool.

## 2 Materials and Methods

### 2.A MPC and Conventional QC

MPC was run daily at morning run‐up on each of the eight Varian TrueBeams in the Author’s department, all running TrueBeam platform software version 2.5. The Linac fleet comprised seven TrueBeams, and one TrueBeam Stx, named G1 to G6 on the main site and Q1 and Q2 at the satellite center. All Linacs were equipped with the aS1200 portal imager, with the TrueBeams equipped with Millennium 120 MLC, and the TrueBeam STx with the HD120 MLC, which were reinitialized monthly in servicing prior to QC. All Linacs can deliver 6 and 10 MV flattened photon beams, and 6, 9, 12, 16, and 18 MeV electron beams, apart from one (G4) which has 6 and 10 MV only.

Additionally, the DailyQA3 (Sun Nuclear Corporation, Melbourne, FL, USA) was used to perform weekly measurements on all beams. The device utilizes a number of ionization chambers and diode detectors to measure output constancy, flatness, symmetry, energy, and radiation field size.^11^

All the parameters tested by MPC and the tolerances are outlined in Table I. The beam measurements were acquired for all beam energies, and the Geometry tests for 6 MV only. Detailed explanations of the methods that MPC employs to measure the various beam and geometric parameters can be found in the published literature.^5^, ^6^, ^7^, ^8^, ^9^, ^10^ There were some occasions where the MPC measurements were not completed due to interlocks during the MPC imaging sequence, for example, when there were Beam Generation Module faults. There were also other occasions where there was a partial set of results, for example, Beam measurements only. All available results were included in the analysis.

**Table 1:** All MPC and comparable Conventional QC test parameters and tolerances.

MPC Group	MPC test parameter	Tolerance	Conventional QC test	Test Method and Equipment	Tolerance
Beam	BeamCenterShift	0.5 mm	–	–	–
	BeamOutputChange	2%	Ionization Chamber Output	Photons: Farmer, 5 cm deep, 95 cm SSD, 10 × 10 field, T41014 stationary water phantom^b^. Electrons: Roos/NACP, dmax, 100 cm SSD, 10 × 10 field, Bart’s Solid Water WTE^c^.	2%
	BeamUniformityChange	2%	Flatness and Symmetry	Octavius 1500 Ionization chamber array^b^ 20 × 20 field. Photons: 5 cm deep, 95 cm SSD. 6/9 MeV: 12 mm, 12/16/18 MeV: 22 mm deep, 100 cm SSD.	3%
Collimation	CollimationRotationOffset	0.5°	Collimator Scale	Marked dot parallel to projected crosshair on gantry rotation.	0.5°
Jaws	JawX1	1 mm	Radiation field size	Ball bearing plate aligned to projected crosshair at 100 cm SSD. MV image acquired. Inspection of position of each jaw edge with respect to images of 1 mm and 2 mm ball bearings on plate. Alternate 10 × 10, 20 × 20 fields monthly.	2 mm
	JawX2	1 mm	Radiation field size		2 mm
	JawY1	2 mm	Radiation field size		2 mm
	JawY2	2 mm	Radiation field size		2 mm
MLC	MLCLeafXX^a^	1 mm	Picket Fence	Inspection of pickets in portal image.	1 mm
	MLCMaxOffsetA	1 mm	Picket Fence		1 mm
	MLCMaxOffsetB	1 mm	Picket Fence		1 mm
	MLCMeanOffsetA	1 mm	Picket Fence		1 mm
	MLCMeanOffsetB	1 mm	Picket Fence		1 mm
Couch	CouchLat	0.7 mm	Couch lateral scales	Move couch known distances with ruler.	1 mm
	CouchLng	0.7 mm	Couch longitudinal scales	Move couch known distances with ruler.	1 mm
	CouchRtn	0.4°	Couch rotation scales	Couch 0°: Marked dot parallel to projected crosshair for couch long movements. Other angles: align plate to crosshair on couch rotation.	0.5°
	CouchVrt	1.2 mm	Couch vertical scales	Move couch known distances with ruler.	1 mm
	RotationInducedCouchShift	0.75 mm	–	–	–
Gantry	GantryAbsolute	0.3^0^	Gantry rotation scales	Gantry leveled with spirit level abutting the Interface Mount at each cardinal angle.	0.5°
	GantryRelative	0.3^0^	Gantry rotation scales		0.5°
Isocenter	IsoCenterKVOffset	0.5 mm	Isoverification kV center	Varian Isoverification	0.5 mm
	IsoCenterMVOffset	0.5 mm	Isoverification MV center		0.5 mm
	IsoCenterSize	0.5 mm	–	–	–

“‐“ indicates that the equivalent conventional QC test was not performed. ^a^Each MLC leaf (XX) is reported individually, for example, leaf 11 is MLCLeaf11. ^b^PTW, Freiburg, Germany. ^c^St Bartholomew's Hospital, London, UK.

The conventional Linac QC testing program followed guidance outlined in AAPM TG142^1^ and IPEM report 81v2,^2^ and was recorded in a QATrack+ v0.2.9.1 database (Multileaf Consulting, Ottawa, Canada). The conventional QC tests and tolerances are in Table I. All tests were performed monthly apart from couch tests and electron flatness and symmetry which were performed every 3 months.

A regular MV and kV panel calibration program was followed, with 3 monthly IsoCalibration, dark field, flood field, and pixel defect map calibrations. Imager QC was performed every 3 months, consisting of distance scaling QC tests for kV and MV panels and kV image quality with the Leeds test object TOR 18FG (Leeds Test Objects Ltd, Boroughbridge, UK).

MPC was baselined in the commissioning period of each Linac once the beam setup was optimized based on Beamscan (PTW, Freiburg, Germany) water tank measurements. There is a function in the MPC application to allow subsequent re‐baselining, required due to drift in MV panel response over time. This will simultaneously set new baseline values for the output, beam uniformity, and beam center. Therefore, on occasions where MPC was re‐baselined to correct the drift in output constancy versus ionization chamber measurement, the beam flatness and symmetry and beam center position were confirmed via conventional QC checks. Ideally, the beam setup would be optimal prior to re‐baselining in order to minimize the systematic errors in the MPC measurement. Due to the pressures of our busy Clinical department, a more practical approach was adopted, whereby we required the conventional QC flatness and symmetry to be less than 2%, and required the conventional QC relating to the beam center position to be within tolerance. The flatness and symmetry level of 2% were set tighter than our suspension level tolerance of 3% (Table I), to reduce the magnitude of any potential systematic error; in the worst‐case scenario, if the symmetry were just less than 2% when baselined, the true symmetry could reach 4% before MPC recorded an out of tolerance result. On implementation of MPC, we regarded these potential systematic errors as acceptable for the daily measurement, having in place weekly DailyQA3 measurements as part of our QC schedule.

There are differences between the methods of MPC measurement of each test parameter and the conventional QC method, Table I. The MPC tolerances are generally the same or slightly less than the conventional QC test tolerances. An example of the differing methods is MPC reports the beam flatness and symmetry as a single uniformity value (this quantity is defined in Barnes and Greer^6^) for the 18 × 18 cm field, while the conventional QC test reports the Varian defined flatness and symmetry values for the PTW Octavius 1500 array measurement of a 20 × 20 cm field. In the following work, we have investigated whether there is concordance between the MPC out of tolerance results and the out of tolerance results reported by the conventional monthly QC program, each following their own respective methods and tolerances.

### 2.B Output constancy

Outputs were measured monthly with ionization chambers and compared with the MPC results for that day for each beam on all Linacs from February 2017 to November 2019. The % difference was calculated as the ionization chamber output–MPC output. Photon outputs were measured with a Farmer ionization chamber and electron outputs with either a Roos or NACP chamber, all traceably calibrated to the UK National Physical Laboratory. The annual field chamber calibrations demonstrated that the absorbed dose to water calibration factors were stable within 0.5% for each chamber.

The local practice was to re‐baseline MPC if the difference between MPC and ionization chamber was >1.5% at the monthly comparison measurement, or if consistently >1% over 3 months. Previous authors^6^, ^9^ have implemented a 1% tolerance on the grounds that the MPC threshold is set in the application at 2%, thus ensuring that daily output is within the TG142^1^ tolerance of 3%. We extended the tolerance slightly on implementation to 1.5% on any given month, and 1% over three consecutive months. We accepted a slightly wider potential discrepancy in the daily MPC reported output, in order to reduce the frequency of MPC calibrations, and required the ionization chamber–MPC output difference to be consistent over several months to avoid incorrectly baselining on an outlying result. We justified this slight increase when MPC was implemented in our department, based on knowledge of the tolerance of ± 5% for output constancy in UK guidance^12^ that was in place at the time (now superseded by report 81v2^2^ which recommends a reduced tolerance of 3%), and the range in tolerances for daily output constancy devices of between ± 1% and ± 5% reported in use in a UK survey of Linac QC.^13^

In order to further evaluate MPC output constancy measurements, we have compared our data with data from a UK national audit of daily 6 MV output measurements.^14^ These output data were acquired from 52 centers, measured with a range of output constancy devices on a range of different Linac models, over a 6‐month period. We have compared the distribution of the 6 MV MPC output data from all Linacs, February 2017–November 2019, with the distribution of measured machine output reported in this UK audit.

In addition, we have assessed the change in MV panel response over time. The 6 MV MPC output results were retrospectively corrected for each MPC re‐baseline event for all Linacs, for comparison with the monthly output results from February 2017 to November 2019. The % difference in the 6 MV absorbed dose to water calibration factor from the annual field chamber cross calibrations of three Farmer field chambers has also been included to indicate the stability of the Farmer ionization chambers.

### 2.C Analysis of MPC records

All the 6 MV Beam and Geometry results from the commencement of treatment on each Linac (ranging from November 2016 for G2 to May 2017 for Q2) until July 2019, a total of 6249 measurements, were analyzed to determine the failure rate of each test parameter, that is, the number of times the given parameter was greater than the MPC defined tolerance. The failures were then classed either true negative or false negative, through cross checking against the machine log book records and QATrack+ to investigate the cause and resolution of any failures. Either the DailyQA3 measurement or corresponding monthly conventional QC tests were performed to investigate the MPC failure. The true negative occurs when MPC finds an out of tolerance parameter that is confirmed as a failure by conventional QC. The false negative occurs when the MPC out of tolerance result is not confirmed by conventional QC.

We limited the review of MPC results to 6 MV Beam and Geometry, and did not review the 10 MV or electron MPC Beam data. This was because the main driver for this study was to validate MPC, to give us sufficient confidence in the MPC results that we could fully incorporate it into our QC program and reduce the frequency of the corresponding conventional QC tests (“Hybrid QC” in Section 4.E.). We have weekly DailyQA3 measurements scheduled in our QC program which provides data on output, flatness and symmetry, field size, and position for all beams, so are not reliant on the MPC results for these measurements. We therefore reviewed the 6MV Geometry tests, the main area of overlap with conventional QC, and the 6MV Beam group which we judged would be representative of the other beam energies.

### 2.D Analysis of Conventional QC records

Further to the analysis of MPC records above, all the monthly and 3 monthly conventional Linac QC test results in QATrack+ from November 2016 until July 2019 on each Linac that were comparable to the checks carried out by MPC were analyzed. These tests and tolerances are in Table I. All the QC records were reviewed and all out of tolerance results investigated and cross checked against the MPC result on the day. This was in order to determine if MPC was sufficiently sensitive to detect the out of tolerance conventional QC results measured during the test period.

### 2.E Estimates of sensitivity and specificity

We have used the MPC and conventional QC data (Sections 2.C and 2.D) to estimate the sensitivity, specificity, and negative predictive value (NPV) of MPC, for the 6 MV Beam and 6 MV Geometry groups of tests. The NPV was estimated from the number of true negative and false negative MPC results recorded from November 2016 to July 2019. A conservative estimate was made for the number of true positives for the estimate of sensitivity. Here, true positives were attributed to monthly QC days only, where we could confidently class positive MPC results as true positive or false positive based on knowledge of the full monthly QC that was performed that day. The specificity was calculated from the conventional monthly QC results recorded from November 2016 to July 2019 attributing true negative if the QC failure was detected by MPC and false positive if MPC did not report an out of tolerance result.

## 3 Results

### 3.A Output records

**Fig. 1:**
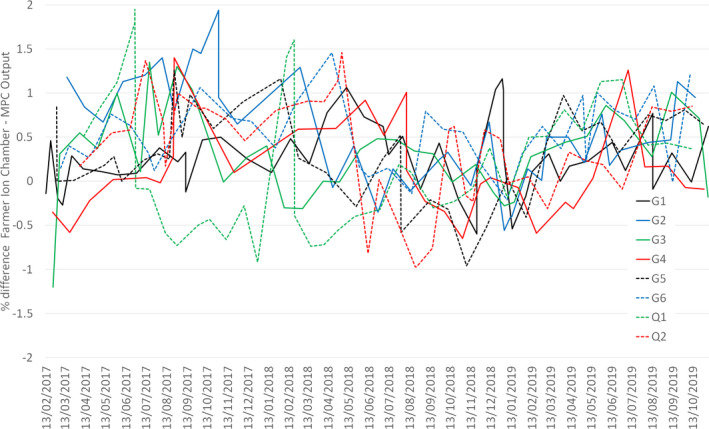
The % difference in 6 MV MPC and ion chamber outputs measured monthly on all eight Linacs, February 2017–November 2019.

Figure 1 illustrates the % difference in 6 MV output as measured by MPC compared with the monthly ionization chamber measurement on all Linacs from February 2017–November 2019. The results for 6 MV are representative of all beam energies. The maximum difference is 2%, with two Linacs demonstrating the largest % differences, Q1 and G2.

The % difference in output as measured by MPC compared with ionization chamber for all the beam energies is summarized in Table II. The mean agreement across all beam energies of the 1933 monthly comparison measurements is 0.2%, with 97.8% of measurements within 1.5%, and 99.7% within 2%. Each beam energy demonstrates similar high levels of agreement between MPC and ionization chamber, apart from 6 MeV which has a slightly lower proportion of MPC results within 1% (85.3%), 1.5% (95.2%), and 2% (98.5%) of the ionization chamber result, and the largest standard deviation of 0.7%.

**Table 2:** The % difference between MPC and ion chamber outputs measured monthly on all eight Linacs.

Beam	Number of measurements	Mean % difference	Min % difference	Max % difference	% within 1%	% within 1.5%	% within 2%	SD
6 MV	306	0.3	−1.2	2.0	87.3	98.7	100.0	0.6
10 MV	308	0.3	−1.5	2.0	89.9	97.7	99.7	0.5
6 MeV	272	0.1	−1.7	2.7	85.3	95.2	98.5	0.7
9 MeV	265	0.1	−1.5	1.8	90.2	98.1	100.0	0.6
12 MeV	267	0.0	−2.9	1.6	91.4	98.1	99.6	0.6
16 MeV	259	0.1	−1.4	1.9	91.1	98.5	100.0	0.6
18 MeV	256	0.1	−1.6	1.6	89.8	98.4	100.0	0.6
	Total	Mean	Min	Max	Total	Total	Total	
	1933	0.2	−2.9	2.7	89.2	97.8	99.7	

**Fig. 2:**
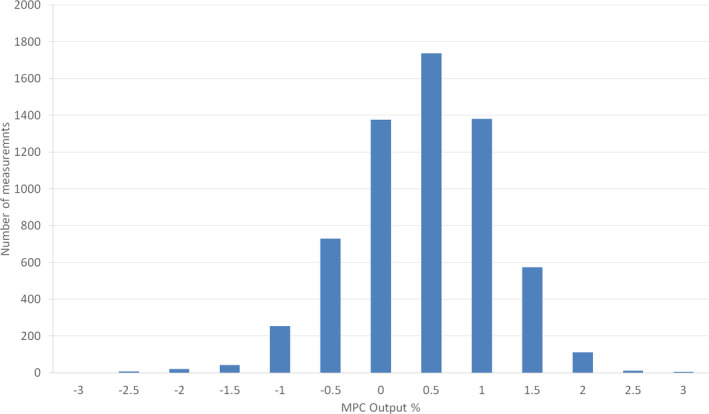
Distribution of MPC measurement of 6 MV output, measured daily on all eight Linacs, February 2017–November 2019.

Figure 2 illustrates the distribution of MPC measurement of 6 MV output, on all eight Linacs, February 2017–November 2019. The data have a mean of 0.2%, and a standard deviation of 0.8% with 16.2% of the MPC outputs exceeding 1% and 0.7% exceeding 2%.

**Fig. 3:**
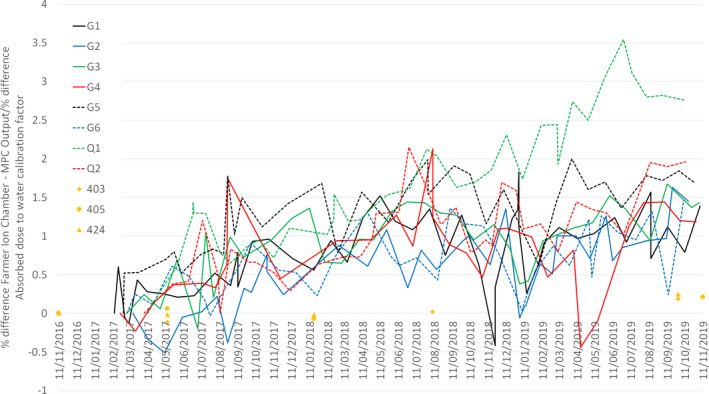
The % difference in 6 MV MPC and ion chamber outputs: measured monthly on all eight Linacs, February 2017–November 2019, corrected for re‐baselining of MPC. The % difference is normalized to zero for the first measurement on each Linac. % difference in 6 MV absorbed dose to water calibration factor: for Farmer chambers 403, 405, 424 determined from annual cross calibrations with the secondary standard. The % difference is normalized to zero for the first cross calibration in November 2016.

Figure 3 illustrates the % difference in 6 MV MPC and ionization chamber outputs measured monthly on all eight Linacs, February 2017–November 2019, corrected for re‐baselining of MPC. The % difference is normalized to zero for the first measurement on each Linac. Over time the MPC measured output reduces relative to the ionization chamber measurement; this reduction in panel sensitivity is generally 0.5–1% per year. The % change in 6 MV absorbed dose to water calibration factor from the initial measurement in November 2016 is also plotted in Fig. 3 to demonstrate the stability of the Farmer chambers throughout this time. The number of output re‐baselining events ranged from 2 to 5 for each Linac over the measurement period, equating to one to two re‐baselines per year.

### 3.B Analysis of MPC records

Table III summarizes all out of tolerance results for the daily 6 MV beam and Geometry MPC test from all Linacs from November 2016 until July 2019. The low number of MPC test failures demonstrates the good overall stability of the Varian TrueBeam Linacs. The highest failure rate of all MPC tests was 0.5% (BeamOutputChange), the next highest was 0.3% (BeamCenterShift), and was 0.1% or less for the remaining parameters. The MPC parameters from Table I that are not listed in Table III all had zero out of tolerance results.

**Table 3:** Summary of all 6 MV Beam and Geometry MPC failures, November 2016–July 2019. Failures are classed FN (False Negative) or TN (True Negative). Failure rate is the total number of out of tolerance MPC results/total number of MPC measurements.

MPC Failure	MPC Tolerance	Linac	Date	Number of MPC results> tolerance	Max Value of MPC parameter	FN/TN	Cause of MPC Failure	Total MPC measurements	Total number> tolerance	Failure Rate %	Total TN	Total FN
BeamOutputChange	2%	Q1	12/11/2017	1	2.04%	FN	Repeated MPC and in tolerance (repeat 1.91%)	6230	33	0.5	18	15
			12/27/2017	1	2.15%	TN	Output drift					
		Q2	10/11/2018	2	2.08%	FN	DailyQA3 — MPC difference					
			10/19/2018	2	−2.35%	TN	Output drift					
			10/23/2018	3	−2.06%	FN	Farmer — MPC difference					
			10/30/2018	1	2.78%	TN	Output drift					
			12/27/2018	1	−2.88%	TN	Output drift					
		G1	12/31/2018	2	−2.16%	FN	Farmer — MPC difference					
			01/02/2019	1	−2.82%	TN	Output drift					
			01/29/2019	4	2.10%	TN	Output drift					
			02/01/2019	1	2.52%	TN	Output drift					
			02/14/2019	1	−3.56%	TN	Output drift					
			02/25/2019	1	−2.25%	FN	DailyQA3 — MPC difference					
		G2	12/18/2017	1	−2.73%	TN	Output drift					
			09/25/2018	1	−2.11%	TN	Output drift					
			10/10/2018	1	−2.05%	FN	Farmer — MPC difference					
			01/02/2019	2	−2.37%	TN	Output drift					
			01/14/2019	1	2.60%	TN	Output drift					
			02/25/2019	1	−2.12%	TN	Output drift					
		G3	08/10/2017	1	−31.61%	FN	Repeated MPC and in tolerance (repeat −0.37%)					
		G4	07/06/2017	4	2.56%	FN	Farmer — MPC difference					
BeamUniformityChange	2%	G3	08/10/2017	1	2.71%	FN	Repeated MPC and in tolerance (repeat 1.08%)	6249	6	0.1	5	1
		G4	06/12/2019	5	4.42%	TN	Mistake in retuning symmetry					
BeamCenterShift	0.5 mm	G2	03/22/2019	9	0.7 mm	TN	Steering error when ion chamber replaced	6246	17	0.3	11	6
		G4	06/13/2019	2	0.53 mm	TN	Mistake in retuning symmetry					
		G5	07/31/2018	6	0.9 mm	FN	MPC Rebaselined, passed Conventional Radiation field size test					
CouchLat	0.7 mm	G1	05/16/2018	1	1.09 mm	FN	Repeated MPC and in tolerance (repeat −0.01 mm)	5012	2	0.0	0	2
		G5	11/29/2018	1	−0.77 mm	FN	Repeated MPC and in tolerance (repeat 0.04 mm)					
RotationInducedCouchShift	0.75 mm	G1	05/16/2018	1	1.16 mm	FN	Repeated MPC and in tolerance (repeat 0.12 mm)	5012	2	0.0	0	2
		G5	11/29/2018	1	0.92 mm	FN	Repeated MPC and in tolerance (repeat 0.08 mm)					
MLCMaxOffset	1 mm	G2	07/10/2017	3	1.67 mm	TN	Resolved by re‐initializing MLC	5944	7	0.1	7	0
		G5	09/27/2018	4	1.05 mm	TN	Resolved by re‐initializing MLC					
IsoCenterKVOffset	0.5 mm	Q1	06/27/2019	1	0.59 mm	TN	X‐ray tube realigned	5147	7	0.1	6	1
		Q2	11/08/2017	1	0.55 mm	FN	Repeated MPC and in tolerance (repeat 0.2 mm)					
		G2	06/07/2019	2	0.69 mm	TN	Post steering adjustment, Isocal performed and corrected					
		G5	01/31/2019	1	0.57 mm	TN	X‐ray tube realigned					
		G6	05/18/2018	2	1.07 mm	TN	X‐ray tube realigned					
IsoCenterMVOffset	0.5 mm	G2	03/22/2019	3	0.92 mm	TN	Post steering adjustment, Isocal performed and corrected	5147	3	0.1	3	0

In total, there are 50 true negative and 27 false negative out of tolerance MPC results. Of the true negatives, MPC detected output drift correctly on 18 occasions, detected a mistake in tuning the beam symmetry due to incorrect water tank setup, and has detected out of tolerance MLC leaves. Re‐initialization of the MLC cleared the out of tolerance MLC leaves. There were three instances when the kV isocenter off‐set test failed, where the x‐ray tube was realigned utilizing the Varian MPC realignment procedure.^15^

Of the 27 false negatives, there are eight occasions where the result was in tolerance on repeat of the MPC. It is not possible to determine the reason in each case definitively, possible explanations are as follows: The repeat measurement for output on Q1 (12/11/2017) is within the inherent repeatability uncertainty (0.06 standard deviation) reported by Barnes and Greer^6^ for MPC output constancy. The large drop in output of −31.6% and uniformity of 2.7% on G3 (08/10/2017) is potentially a measurement error by MPC. Since MPC is repeatable to within 0.05 mm or 0.04 degrees for couch and imaging,^7^ this does not account for the other instances. The in tolerance repeat results for couch lateral and rotation induced couch shift for G1 (05/16/2018) and G5 (11/29/2018) and the Isocenter kV offset for Q2 (11/08/2017) is likely a result of the new setup correcting a slight setup error of the MPC phantom in the stand.

There were a further 13 occasions where the measurement of output with an alternative device (either Daily QA3 or ionization chamber) was within tolerance, demonstrating that the MPC output result was a false negative. This occurs due to allowing a difference of up to 1.5% between MPC and ionization chamber measurements when the MPC output calibration is checked during monthly QC. The remaining six false negatives are all out of tolerance BeamCentreShift results for the G5 Linac. Here, the conventional QC indicated no issues with the beam alignment: The crosshair walkout and light field jaw settings were < 0.5 mm and < 1 mm, respectively, and were consistent with previous months, and the Radiation field size QC indicated no issues with beam position. Therefore, MPC was subsequently re‐baselined.

### 3.C Analysis of Conventional QC records

Table IV summarizes all the occasions where the conventional QC tests that test equivalent parameters to MPC have failed during the 238 monthly QCs in the period from November 2016 until July 2019. The remaining conventional QC tests from Table I that are not listed in Table IV all had zero out of tolerance results. We found that MPC detected all the conventional QC failures, with an out of tolerance result for the relevant test, with the exception of some ionization chamber output and radiation field size tests. MPC correctly detected the QC failures for output, radiation field size, beam symmetry, and the Isoverification kV and MV center tests. There were some occasions where the ion chamber measurement was out of tolerance, but MPC reported the output in tolerance. As discussed in Section 3.B., this occurs due to allowing a difference of up to 1.5% between MPC and ionization chamber measurements when the MPC output calibration is checked during monthly QC. The occasions where conventional QC reported the radiation field size out of tolerance are likely attributed to the misalignment of the radiation field size phantom, since the repeat conventional QC measurement was in tolerance.

**Table 4:** All Conventional QC test failures equating to parameters tested by MPC, November 2016–July 2019.

Conventional QC Test	Tolerance	Linac	Date	Cause of QC Test Failure	Detected by MPC
Ionization chamber Output	2%	Q1	27/12/2017	Output drift	Yes
		Q2	28/04/2018	Output drift	No^a^
		Q2	30/10/2018	Output drift	Yes
		Q2	27/12/2018	Output drift	Yes
		G1	16/03/2018	Output drift	No^a^
		G1	09/05/2019	Output drift	No^a^
		G2	03/01/2019	Output drift	Yes
		G2	14/01/2019	Output drift	Yes
		G4	23/11/2017	Output drift	No^a^
		G4	09/08/2018	Output drift	No^a^
		G5	28/05/2019	Output drift	No^a^
Radiation Field Size	2 mm	G2	17/03/2017	QC test passes on following month	No^b^
			03/05/2019	During period with Steering error	Yes
		G5	27/03/2017	QC test passes on following month	No^b^
		G6	20/02/2017	QC test passes on following month	No^b^
			25/04/2017	QC test passes on following month	No^b^
Isoverification kV center	0.5 mm	G2	07/06/2019	Replacement ion chamber, Steering adjustment	Yes
Isoverification MV center	0.5 mm	G2	07/06/2019	Replacement ion chamber, Steering adjustment	Yes
6 MV Symmetry PTW Array	3%	G4	13/06/2019	Mistake in retuning symmetry	Yes

MPC detects all the failures, with the exceptions of: ^a^Occasions where the ion chamber measurement is out of tolerance, but MPC reports in tolerance due to the MPC–ion chamber output tolerance of 1.5%. ^b^Occasions where it is probable that the conventional QC falsely reported the incorrect field size (likely due to misalignment of the phantom), since the repeat conventional QC measurement is in tolerance.

### 3.D Estimates of sensitivity and specificity

The estimates of the sensitivity, specificity, and NPV for the 6 MV Beam group (output, uniformity, and center shift) and for the 6 MV Geometry group are in Table V. They are calculated from the MPC and conventional QC data in Tables III and IV. These are conservative estimates, and the true values of sensitivity are likely to be much closer to 100% than these values.

**Table 5:** Estimates of sensitivity, specificity, and NPV for the 6 MV Beam and Geometry test groups.

MPC Test Group	Sensitivity	Specificity	NPV
6 MV Beam	93%	54%	61%
6 MV Geometry	98%	100%	76%

## 4 Discussion

### 4.A Output constancy

The MPC versus ionization chamber output constancy results presented in this work are similar to the agreement reported by previous authors.^6^, ^9^ The mean agreement in the monthly MPC output compared with ionization chamber measurements was 0.2% across all beam energies, with means ranging from 0.0 to 0.3 % for each energy, in agreement with Binny et al^9^ who demonstrated that mean output variation was within ± 0.5% compared with the Farmer ionization measurements.

In general, we found the agreement between MPC and ionization chamber to be good, with 97.8% of all measurements within 1.5%. It was found that the least stable energy was 6 MeV, with a slightly lower proportion of MPC measurements within 1.5% (95.2%) compared with the other energies and a wider standard deviation of 0.7%. This is possibly due to the inherent instability of this beam energy, our local experience is that 6 MeV has the least stable unservo’d dose rate, requiring more regular tuning by our engineers. Although we found the MPC–ionization chamber agreement for 6 MeV to be less consistent than the other energies, it was considered acceptable locally.

We report larger maximum differences between MPC and ionization chamber outputs than have been reported previously,^6^, ^9^ with maximum % differences of 2.7% for 6 MeV and 2.9% for 12 MeV and a maximum difference of 2% for the remaining energies, Table II. This will be partly due to allowing a tolerance of up to 1.5% in the MPC to ionization chamber output comparison measurement compared with the tighter 1% tolerance.^6^, ^9^ The larger difference may also be attributed to investigating the output stability over a longer time frame and on a larger number of Linacs. Barnes and Greer^6^ report a much smaller maximum difference of 0.6%, but this was measured on a single Linac over a 5‐month period, while we assessed output constancy for just under 3 yr on eight Linacs. The comparison of MPC and ionization chamber output for six TrueBeams over periods ranging 4.5–12 months by Binny et al^9^ reports the mean data only. A subset of the data that is graphed in the publication by Binny et al^9^ indicates maximum differences of 1–1.5% for some MPC and ionization chamber output comparisons, but it is not possible to compare further since the full data are not available.

The maximum dose differences of up to 2.9%, and the proportion of measurements within 1%, 1.5 %, and 2% (Table II) demonstrate that MPC is suitable for a daily output constancy check, but not for the measurement of absolute output. We would advocate the use of an independent check device, such as the Daily QA3 on a weekly basis, in accordance with previous recommendations,^9^ and a periodic ionization chamber check of MPC calibration.^6^, ^9^

The % difference in the monthly MPC and ion chamber outputs, Fig. 1, demonstrates improved agreement over the course of the data acquisition. A contributory reason for this is may be that the Linac Monitor chambers were diagnosed to be faulty and replaced on G1, G2, and Q2 in April 2019, March 2019, and January 2019, respectively. It is possible that a smaller undiagnosed leak in the Monitor chamber was present in the months before changing them, which contributed to larger differences in the MPC and ion chamber outputs measurements, whereby the Linac output had changed between the MPC measurement and the ion chamber measurement.

### 4.B MPC–Ionization Chamber Output test tolerance and frequency

There are a number of factors to be considered when setting the MPC v ionization chamber output tolerance and calibration check frequency, including the rate of Linac output drift, the reduction in panel sensitivity over time, the agreement required for the daily output check, and the frequency of MPC recalibrations.

Our local data demonstrate an increase in output of between 3 and 4% per year for our eight Linacs over the first 3 yr of Linac use. This is in agreement with the data from Barnes and Greer^6^ which suggests an increase of 3% per year for 6 MV output measured with ionization chamber for the TrueBeam, and also the published literature for previous Varian Linac models. Hossain^16^ reports an increase in output of 2–4% per year on 1 Trilogy and 2 iX Varian Linacs, and Grattan and Hounsell^17^ report an increase in output of 3% per year for the Varian 2100C/D accelerator over the first 4 yr of use, then a decrease of 0.4% per year for the following 3 yr.

The drift in MPC response over time has been previously reported,^6^, ^9^ and has been attributed to gradual changes in the panel sensitivity. This is demonstrated in the small positive offset of 0.2% in the mean agreement of ionization chamber–MPC output difference, Table II. Since the panel sensitivity reduces over time, this difference will tend to be positive. From Fig. 3, we were able to estimate the reduction in MV panel sensitivity to be 0.5–1% per year, which is consistent with the drift of 0.5% over 5 months in Barnes and Greer.^6^ In addition, there are data in the literature for the long‐term stability of the Varian portal imagers with respect to output constancy where this has been studied independent of MPC. These generally indicate no or minimal long‐term drift, contrary to our findings, and those of Barnes and Greer.^6^ Sun et al^4^ report no observed drift for the aS1000 portal imager over 6 months, although it is possible that this is in agreement with our findings, since we would estimate a change in sensitivity of 0.25–0.5% over this period, while they report the portal imager output constancy measurement to be within 0.5% compared against ion chamber. King et al^18^ report that the change in aS500 portal imager response is less than 0.5% for 6 and 18 MV output constancy on three Linacs over 3 yr, and Greer and Barnes^19^ report the variation in aS500 portal imager response to have a standard deviation of 0.4% for 6 and 18 MV output constancy over 7 months. These observed differences may be due to differences between the aS1200 and aS500 panels. We also found there to be a slight variability in the change in panel sensitivity between machines, likely due to varying MV panel usage for each Linac. Overall, the panel demonstrates sufficient long‐term stability to be useful for daily monitoring of output.

This level of drift in panel sensitivity over time of 0.5–1% per year would indicate that the frequency of ionization chamber checks of MPC output calibration could be 3 monthly as proposed by Binny et al,^9^ rather than monthly as suggested by Barns and Greer.^6^ Despite this, we compare MPC v ionization chamber on a monthly basis since we have the results already available for comparison, as MPC is run on every monthly QC day as part of routine run‐up. This approach enables us to check that any MPC–ionization chamber difference is consistent over 3 months prior to any calibration and ensures we do not unnecessarily calibrate MPC on occasions of measurement noise. The month to month variability in ionization chamber–MPC output measurements for 6 MV can be seen in Fig. 3, with occasional differences of up to 1% from the overall upward trend. These occasions are likely to be a result of the random measurement noise, from the MPC output and the ionization chamber measurement, and also from the change in machine output between the MPC measurement at run‐up and the output measurement later in the day (measured locally to be up to 0.5%).

The 6MV MPC output data from all eight Linacs, February 2017–November 2019, Fig. 2, has a comparable distribution to the UK national audit of daily 6 MV output measurements,^14^ both are normally distributed with a standard deviation of 0.8%. The MPC data compare favorably, with 16.2% of the MPC outputs exceeding 1% and 0.7% exceeding 2%, compared with the national audit data^15^ where 24.3% of output measurements exceed 1%, and 1.9% exceed 2%. This confirms the appropriateness of MPC as an output constancy device and the tolerances employed in this study.

The choice of MPC versus ionization chamber output tolerance will be a balance of increased accuracy in MPC output against the increased frequency of calibrations. We are now considering tightening our tolerances based on this work, from 1.5% and 1% over three consecutive months, to 1% and 0.7%. Referring to Table II, at least 85% of results are within 1% for each energy, suggesting the tolerance level could be reduced without incurring an excessive number of additional MPC calibrations.

This tightening of the output tolerance would be expected to impact the results of this study favorably; the difference in the monthly ionization chamber and MPC output measurements reported in Table II would be reduced. Similarly, it would reduce the frequency of false negative and false positive MPC output measurements, and therefore increase the NPV, sensitivity, and specificity values of the 6 MV Beam group, Table V.

### 4.C Estimates of sensitivity and specificity

This work has built on the published literature which has demonstrated MPC’s ability to detect deliberate faults,^6^, ^7^, ^8^, ^10^ by demonstrating that MPC is capable of detecting real‐world Linac faults in a clinical setting. Over the test period, MPC has demonstrated its value in detecting true out of tolerance results on multiple occasions across the fleet of Linacs. There were 50 true negatives recorded, outlined in Section 3.B. Similarly, the analysis of all conventional QC over this same period indicated that MPC picked up all faults that were detected by conventional QC, with the exceptions noted in 3.C.

Estimates of the sensitivity, specificity, and NPV for the 6 MV Beam group (output, uniformity, and center shift) were 93%, 54%, and 61%, and for the 6 MV Geometry group were 98%, 100%, and 76%. These are conservative estimates. The true values of sensitivity are likely to be much closer to 100% than these values. We could only be absolutely confident in the nature of the positive MPC result on monthly QC days only, so chose to include only these days, rather than estimate based on all MPC measurements over the 3‐year acquisition period. The specificity of the 6 MV Beam group is low due to the occurrence of false positives where MPC does not detect the out of tolerance output, due to allowing a difference of up to 1.5% between MPC and ionization chamber measurements for the monthly check of MPC output calibration. This effect similarly impacts the NPV, with occurrences of false negatives due to the 1.5% MPC output calibration tolerance. It is not possible to eliminate all false positives and negatives, due to the inherent instability of the MV panel output measurement, but the absolute number could be reduced by reducing the MPC output calibration tolerance. The low NPV for the 6 MV Geometry group of 76% is due to false negatives that have arisen due to setup of the MPC phantom. On these occasions, MPC has passed on repeat measurements with a new setup. Despite the low specificity and NPV, we find it acceptable given the low failure rate for the 6 MV Beam and Geometry test. This is less than 0.5% for individual test parameters, so we do not find it overly burdensome to investigate MPC failures. In order to mitigate the effect of false negatives, on occasion that the daily MPC fails, we repeat the MPC measurement with a new setup, and if there is a repeated beam measurement failure, we measure output with an independent device — the DailyQA3. We have found that this approach works effectively in the clinical department, and have found MPC to be an extremely valuable QC tool.

### 4.D Limitations

A potential weakness of this study comparing MPC and conventional QC testing is that we are not able to make direct comparisons between tested parameters since the testing method and tolerances may differ between MPC and conventional QC. Despite this, we have successfully taken a high‐level comparison, demonstrating the correlation in detection of real‐world faults by the separate MPC and conventional QC systems with their own procedures and tolerances.

It should be noted that we have experienced some reliability issues with MPC during the 3 yr that it has been in use in our department. There have been occasions whereby the Linac produces an interlock during MPC delivery (usually a Beam Generation Module fault interlock) and is unable to complete the MPC imaging sequence. This behavior is not seen during normal treatment delivery. Our hospital Engineering team have worked closely with Varian to resolve this, and we can report that since spring 2019, MPC has consistently run without producing these interlocks on all the Linacs. There was no specific repair made to the machines, but contributing improvements were upgrading from 2.5 MR1 to 2.5 MR2 which had less stringent internal interlock tolerances (each Linac was upgraded from February 2017 to April 18), setting the Automated Frequency Control coefficient to a Varian recommended standard value, implementing regular monthly beam tuning of gun and RF, and reconnecting or replacing cables in the beam generation line.

### 4.E “Hybrid QC”

There is a growing trend in the published literature of MPC replacing conventional QC. Barnes and Greer^5^ have replaced the daily checks using Daily QA3 with MPC beam constancy checks, and later reported that the MPC Geometric tests were suitable for daily QC.^6^, ^7^ Binny et al^8^ have also verified the capability of MPC for daily output and uniformity tests. Li et al^9^ also report that they have incorporated MPC into daily QC, replacing previous testing methods, and state that MPC also has the potential to replace monthly QC checks. As a result of our 3 yr of experience of daily MPC measurements, we are now in the process of reviewing the frequency of conventional monthly QC tests in our QC program. Conventional ionization chamber output measurements are retained monthly due to the variation we have experienced between MPC and ionization chamber output, in line with that reported elsewhere.^9^ We are replacing many of the conventional geometric monthly QC tests with the daily 6 MV Beam and Geometry MPC test. The corresponding monthly conventional tests are now carried out on an annual basis, and in response to specific faults and repairs, acting as an ongoing independent check of MPC. We have adopted the term “Hybrid QC” for this approach, a hybrid of conventional and automated QC methods. This Hybrid QC method utilizes the benefits of automated QC of reduced operator error, increased accuracy, and time savings, but avoids complete reliance on automation. This approach could be adopted by other centers using Varian TrueBeam Linacs, freeing up treatment time and resources so the medical physicist can focus on other areas like development activities. We would advocate thorough commissioning of MPC prior to using it in place of conventional QC tests.

## 5 Conclusions

The variation in output as measured by MPC versus ionization chamber measurement indicates that MPC is appropriate as a daily output constancy check, but cannot replace monthly ionization chamber output measurements. Our comparison of the MPC and conventional QATrack+ records has provided evidence that MPC is a robust and sensitive method of performing beam and mechanical checks in a clinical setting. There were a small number of false negative results reported by MPC, and we would advocate the use of independent methods, such as use of the Daily QA3 device, to quickly resolve these when they occur. We are in the process of re‐evaluating the frequency of our monthly geometric conventional QC tests with a view to reducing the frequency due to our confidence in MPC.

## Conflict of Interest

The authors have no relevant conflicts of interest to disclose.
